# How Heavy Is an Illusory Length?

**DOI:** 10.1177/2041669516669155

**Published:** 2016-09-21

**Authors:** Anouk J. de Brouwer, Jeroen B. J. Smeets, Myrthe A. Plaisier

**Affiliations:** Vrije Universiteit Amsterdam, The Netherlands; Queen’s University, Kingston, ON, Canada; Vrije Universiteit Amsterdam, The Netherlands

**Keywords:** heaviness perception, size-weight illusion, Müller–Lyer illusion, multisensory perception

## Abstract

The perception of object properties, such as size and weight, can be subject to illusions. Could a visual size illusion influence perceived weight? Here, we tested whether the size-weight illusion occurs when lifting two physically identical but perceptually different objects, by using an illusion of size. Participants judged the weight and length of 11 to 17 cm brass bars with equal density to which cardboard arrowheads were attached to create a Müller–Lyer illusion. We found that these stimuli induced an illusion in which the bar that was visually perceived as being shorter was also perceived as feeling heavier. In fact, a 5-mm increase in illusory length corresponded to a decrease in illusory weight of 15 g.

Could an illusion in one sensory modality cause a chain of subsequent illusions in other sensory modalities? Here, we show that an illusory visual size difference can trigger an illusory haptic weight difference.

In the size-weight illusion, a small object is perceived to be heavier than a larger object of the same mass (Charpentier, 1891). Would this illusion occur for identically sized objects when the perceived size is manipulated using a visual illusion? To answer this question, we used the well-known Müller–Lyer illusion. In this visual illusion, a line appears shorter or longer, depending on the direction of the arrowheads flanking the ends of the line.

Previous research has shown that the size of an object is used to estimate the mass of the object and scale lift forces (Gordon, Forssberg, Johansson, & Westling, 1991). Brenner and Smeets (1996) have shown that when lifting a disk in the Ponzo illusion, the lift forces are scaled according to the illusory size of the object. This suggests that a visual size illusion influences the anticipated mass of an object, which might induce a size-weight illusion. On the other hand, it has been reported that perceptual and sensorimotor predictions are independent (Flanagan & Beltzner, 2000).

Thirteen participants performed two conditions in which they either judged the weight (by lifting) or length (by looking) of three-dimensional Müller–Lyer stimuli. Each stimulus consisted of a black brass bar (1.4 × 1.4 cm thick) to which black cardboard arrowheads were attached, so that the arrowheads moved with the bar when lifted. Participants were seating at a table and were presented with two Müller–Lyer stimuli at a time (Figure 1): a standard stimulus (14 cm, 231 g) with inward or outward pointing arrowheads, and a test stimulus (11–17 cm, 181–279 g) with opposite arrowheads. In the weight condition, participants verbally indicated which of the two stimuli felt heavier after sequentially lifting the stimuli. In the length condition, participants indicated which of the two stimuli appeared longer by looking at the two stimuli. We used bars of equal density (8.4 g/cm^3^), so the weights of the stimuli co-varied naturally with the lengths. The test stimulus that physically matched the standard in size (i.e., the 14 cm bar) also matched the standard in weight. The same stimuli were used in the length and weight condition. To reduce possible interference of the length condition on the weight condition, the conditions were performed in separate sessions, with the weight condition being performed first.

**Figure 1.**
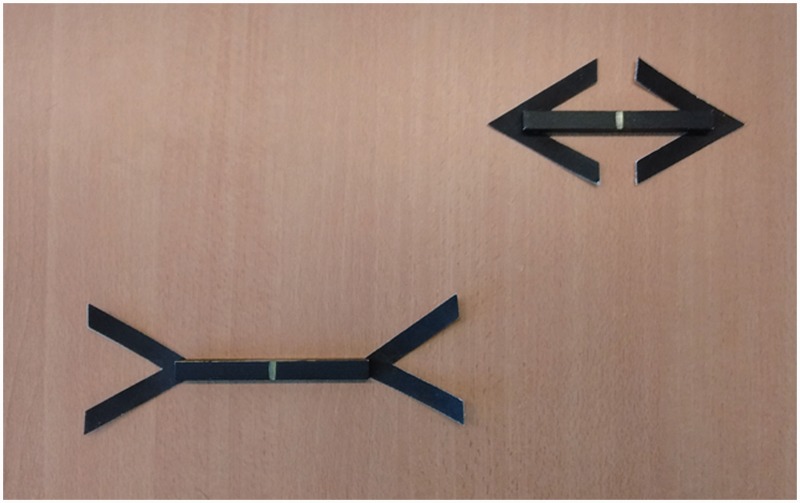
The two configurations of the stimuli. Top-right: “short,” bottom-left: “long.” The position and configuration of the standard stimulus were counterbalanced over the top-bottom and left-right position. In the length condition, participants only viewed the stimuli. In the weight condition, participants were instructed to grasp and lift each bar at its center as indicated by the small line.

For both conditions, we measured a psychometric curve for each standard stimulus (“short” and “long”). We used a method of constant stimuli in which each combination of standard stimulus and test stimulus (*n* = 22) was presented nine times. Psychometric curves were fitted using maximum likelihood estimation (Wichmann & Hill, 2001) to obtain the point of subjective equality (PSE) for each participant and condition. The shift in PSE was calculated as the PSE for the “long” standard stimulus minus the PSE for the “short” standard stimulus and divided by two (note that participants were always comparing two illusions). The illusion effect is the PSE shift expressed as a percentage of the length or weight of the standard stimulus. One participant was excluded because the PSE for the weight session was outside of the stimulus range.

Figure 2(a) shows that the psychometric curves, and thus the PSEs, were shifted with respect to each other. On average, the “short” stimulus had to be 5 mm (4%) longer than the “long” stimulus (one-sample *t* test: *t*(11) = 3.86, *p* = 0.003) to be perceived as having the same length (Figure 2(b)). Furthermore, the “short” stimulus had to be 9 mm shorter (i.e., 15 g or 7% lighter; one-sample *t* test *t*(11) = 5.54, *p* < 0.001) than the “long” stimulus to be perceived as having the same weight, reflecting a size-weight illusion. This means that the same object that was perceived to be 5 mm longer in the length condition was perceived to be 15 g lighter in the weight condition.

**Figure 2.**
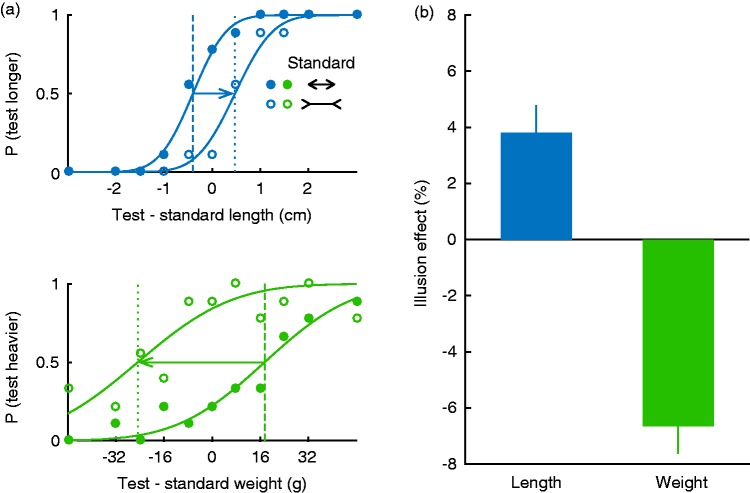
Results. (a) Psychometric curves of an example participant. The arrows indicate the PSE shift between the “short” and “long” standard stimuli. (b) Illusion effects: Half of the PSE shift (“long”−“short” standard) expressed as percentage of the length or weight of the standard stimulus, averaged across participants with the associated standard error.

We tested whether a size-weight illusion would occur for objects of identical physical size and weight, but different perceptual sizes due to the Müller–Lyer illusion. Our results show that we were able to induce a visual illusion by adding arrowheads to three-dimensional bars. When lifting these bars, these arrowheads induced a change in the perceived weight: perceptually longer stimuli were perceived as lighter than perceptually shorter stimuli. Since we did not alter the natural size-weight relationship of the test stimuli, this weight illusion can only be the result of the arrowheads inducing a visual length illusion.

Surprisingly, the decrease in perceived weight did not match the decrease in weight that would be expected given the visual perceived length difference. In a previous study, we found that a *physical* size increase of 20% caused a perceived weight reduction of 5.5% (Plaisier & Smeets, 2015). Here, we found a much larger effect: a 4% increase in *illusory* length corresponded to a 7% decrease in perceived weight. A possible explanation for this discrepancy is that participant’s sensitivity to the Müller–Lyer illusion and size-weight illusion might be different (Kawai, Henigman, MacKenzie, Kuang, & Faust, 2007). Another possibility is that participants use different size estimates when judging length then when judging weight (Smeets, Brenner, de Grave, & Cuijpers, 2002). For example, we have shown that in the size-weight illusion, size can influence the weight percept independent of the volume of material (Plaisier & Smeets, 2015). Thus, perceived weight might, for instance, also be influenced by the fact that total length of the stimulus, including the fins, is much larger in the visually long illusion than in the short illusion.

Summarizing, we show that the same visual illusion that caused a 5-mm illusory length increase caused an illusory weight decrease of about 15 g.
